# An appropriate ratio of unsaturated fatty acids is the constituent of hickory nut extract for neurite outgrowth in human SH‐SY5Y cells

**DOI:** 10.1002/fsn3.1623

**Published:** 2020-11-12

**Authors:** Fei Gao, Jianfeng Wu, Yu Zhou, Jianqin Huang, Jidong Lu, Yongchang Qian

**Affiliations:** ^1^ Traditional Chinese Medicine Unit School of Forestry and Biotechnology Zhejiang A&F University Hangzhou China

**Keywords:** hickory nuts, neurite outgrowth, neurotrophic factors, ratio of UFAs, SH‐SY5Y cells, unsaturated fatty acids

## Abstract

Hickory nuts (*Carya cathayensis* Sarg, CCS), a well‐known Chinese medicinal nut, is thought to improve memory in Chinese folks. However, functional constituents have not been scientifically identified. In this study, human SH‐SY5Y cells, combined with Q‐TOF mass spectrometry (Q‐TOF‐MS) and standard substances, were used to evaluate the function in neuronal development and to identify constituents of CCS hydrophobic extracts (CCS‐HE). Data showed that CCS‐HE but not the control induced neurite outgrowth of SH‐SY5Y cells in a dose‐dependent manner, supported by which CCS‐HE induced the expression of nerve growth factor (NGF), neurofilament 160 (NF160), and neuronal peptide Y (NPY) mRNA. Q‐TOF‐MS analysis with standard substances indicated that linolenic acid (LNA), linoleic acid (LA), and oleic acid (OA) were the main constituents in CCS‐HE. Furthermore, mixtures of these unsaturated fatty acids (UFAs) at the natural ratio (1:8:16) significantly induced neurite outgrowth and gene expression of NGF, NF160, and NPY in a dose‐dependent manner. However, the individual and alternative ratios were not effective to induce the neurite outgrowth and gene expression of NGF, NF160, and NPY. These data implicate that an appropriate ratio of UFAs is the main constituent for the neurite outgrowth.

## Graphical abstract

Hickory nuts (*Carya cathayensis* Sarg, CCS), a well‐known Chinese medicinal nut, is thought to improve memory in Chinese folks. Our results implicate that an appropriate ratio of UFAs is the main constituent for the neurite outgrowth.

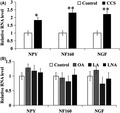

## 1 INTRODUCTION

*Carya cathayensis* Sarg (CCS) has been used as a conventional folk medical nut for health care in China for a long time. In the “Bencao Gangmu,” an ancient Chinese book on herbal medicine, it was described that the pecan nuts of CCS were beneficial for brain and kidney health. Traditionally, one of the main benefits of CCS was considered to help our brain by improving the memory ability and delaying the neuronal degeneration. Current nutrition research has indicated that CCS contains a variety of nutrients, including high levels of unsaturated fatty acids (UFAs) that are supported by the harmonious expression of key ones for UFAs (Huang et al., 2016). It also contains saturated acids, vitamins, and trace elements. Studies show that constituents from the leaves of CCS play a role in antioxidation, antitumor, and inhibition of angiogenesis induced by vascular endothelial growth factor (VEGF) (Cao et al., 2012; Tian et al., 2014). However, there is no desired model to evaluate the function of CCS in neuronal development and identify the bioactive constituents of CCS.

Neurite outgrowth is an important event in neuronal path finding and establishment of synaptic connections during development of nervous system (Barnes & Polleux, 2009; Cheng & Poo, 2012). It is also essential in neuronal plasticity and neuronal regeneration after injury (Chen, Yu, & Strickland, 2007; Huang & Reichardt, 2001; Loers & Schachner, 2007) and neurodegenerative conditions, such as Alzheimer's and Parkinson's diseases (De Vos, Grierson, Ackerley, & Miller, 2008; Goldberg & Barres, 2000). Therefore, treatments aiming at promoting neurite outgrowth and preserving the neurite network and synaptic connections for recovery are needed. Neurofilaments (NFs) are intermediate filaments in neurons that are considered to add rigidity, tensile strength, and possibly intracellular transport guidance to axons and dendrites. Neurofilament (NF) proteins, including NF200, NF160, and NF58, have two fundamental functions, supporting axonal structure and protecting neuronal survival and neurite outgrowth (Al‐Chalabi & Miller, 2003). Nerve growth factor (NGF) is a classic growth factor in family of neurotrophic factors, a group of proteins that are mainly synthesized and secreted by neurons and astrocytes and that are crucial for neuronal survival, growth, and differentiation (Sofroniew, Howe, & Mobley, 2001). Studies have shown that NGF causes axonal branching and elongation (Madduri, Papaloizos, & Gander, 2009) and NGF could induce the expression of NF proteins (Huang et al., 2010; Schimmelpfeng, Weibezahn, & Dertinger, 2004). In contrast, NGF defect results in an undergoing apoptosis in neurons (Freeman et al., 2004). NGF is also reported to play a potential role in depression and schizophrenia (Martino et al., 2013; Martinotti et al., 2012). Neuropeptide Y (NPY) is a 36 amino acid peptide and extensively distributes in neurons of the central and peripheral nervous systems (Eipper, Stoffers, & Mains, 1992). It is found that NPY has a higher level than do all other peptides studied in mammalian brain (Gray & Morley, 1986). It acts as another neurotransmitter and/or another modulator of several neuroendocrine functions (Colton & Vitek, 2006). Studies have proved that NPY could exert neuroprotection against Aβ toxicity in both neuroblastoma and primary cells, function as a neuroprotective agent against AD, and indirectly induce neurite outgrowth (White & Mansfield, 1996). Studies reported that NPY exerted its neuroprotective roles by influencing the gene expression of neurotrophins (Angelucci et al., 2014; Croce et al., 2011) and by inducing the neurite outgrowth (White, 1998).

Human SH‐SY5Y neuroblastoma cell line has extensively used as a neuronal model for studies of neurochemistry, neurobiology, and neurotoxicology (Nciri et al., 2014; Qian, Zheng, & Tiffany‐Castiglioni, 2009; Wen et al., 2013; Yang, Sheng, Sun, & Lee, 2011). In this study, we used SH‐SY5Y cell line as a neuronal model to evaluate the function of bioactive constituents of CCS hydrophobic extracts (CCS‐HE). The neurotrophic property of CCS‐HE in stimulation of neurite outgrowth and in gene expression of NGF and NFs was validated. The possible involvement of NPY was also determined to further reveal the mechanism of neurites outgrowth by CCS‐HE. In addition, an appropriate ratio of LNA, LA, and OA was identified as the main bioactive constituent of CCS‐HE in the induction of neurite outgrowth. This study provides the information on which human SH‐SY5Y cells would be a desired model to evaluate the function of CCS‐HE in neuronal development by measurement of neurite outgrowth.

## 2 MATERIALS AND METHODS

### 2.1 Preparation of CCS hydrophobic extracts (CCS‐HE)

Fresh hickory nuts that had been frozen in liquid nitrogen were ground into powder. Ten grams (10 g) of powder were used to extract hydrophobic constituents with 150 ml of petroleum ether in Soxhlet apparatus for 6 hr. After extraction, the petroleum ether was thoroughly removed on a rotary evaporator and CCS hydrophobic extracts (CCS‐HE), approximately 5 g, were stored at −80°C. CCS‐HE was dissolved in fresh DMSO to prepare a stock concentration of 160 mg CCS‐HE/ml and filtrated with a 0.45 µm membrane. To minimize the cytotoxicity of DMSO to SH‐SY5Y cells and maximize CCS‐HE solubility in cell culture medium, CCS‐HE stock solution was diluted in 1:400 or larger dilution in the medium and ultrasonic was used to promote CCS‐HE solubility, generating a concentration of 0.25% (v/v) DMSO that had no influence on the growth of SH‐SY5Y cells in pre‐experiment and a highest final concentration of 0.4 mg/ml CCS‐HE in the medium.

### 2.2 Cell culture

Human SH‐SY5Y cells were purchased from ATCC, and SH‐SY5Y‐EGFP cells were gifted from NUPTEC, respectively. Both cells were maintained in T‐25 flasks (Falcon) with DMEM/F12 medium (Sigma) containing 10% fetal bovine serum (FBS, Invitrogen) under conditions of 5% CO_2_ and 37°C. The medium was changed every other day. For experiments, cells were cultured in 60‐mm culture dishes (Corning) at an initial density of 50,000 cells/ml.

### 2.3 Treatment with CCS‐HE or standard substances

In order to measure effects of CCS‐HE or standard substances including linolenic acid, linoleic acid, and oleic acid on neurite outgrowth, CCS‐HE at the final concentrations of 0, 0.1, 0.2, and 0.4 mg/ml or standard substances in an appropriate ratio of linolenic acid, linoleic acid, and oleic acid were added to the cell culture medium for 6 days and the concentration of DMSO in each treatment were adjusted to 0.25% (v/v). Twenty ng/ml of bFGF was used as a positive control for neurite outgrowth (Boku et al., 2013). To determine gene expression of neurofilament 160 (NF160), NGF, and NPY, CCS‐HE was added to cell culture medium as same concentrations as mentioned above for 24–48 hr. To better visualize the neurite outgrowth under a fluorescent microscope, the ratio of SH‐SY5Y cells to SH‐SY5Y‐EGFP cells was 6:1.

### 2.4 Measurement of neurite length

Cells were cultured as described above in 6‐well plates, and six images of live cell morphology were randomly captured for each treatment on a Leica fluorescent microscope equipped with Compix Imaging System. The images were labeled with a scale in proportion to the magnification, leading to a calculation of μm/pixel. Each cell had several neurites with different lengths. Usually, the neurite length was measured for total length of all neurites in single cell or for longest neurite length of each cell. In this study, the longest neurite of each cell was randomly selected to measure the neurite length and 50 longest neurites were measured from six images of each treatment. The actual length of neurite outgrowth was calculated as follows: measured pixel × μm/pixel. Alternatively, the neurite lengths from different treatments were compared by percentages of cells with a longer than 40 μm neurite length over total 50 selected cells.

### 2.5 Real‐time PCR

Total RNAs were extracted from cells using the TRIzol method (TaKaRa) according to the manufacturer's instructions. The quality and quantity of total RNAs were assessed by using the NanoDrop ND‐1000 spectrophotometer, and their integrity were tested by 1% agarose gel electrophoresis. Reagents of the PrimeScript II 1st strand cDNA Synthesis Kit (TaKaRa) were used in total cDNA reverse transcription (RT) according to the manufacturer's protocol. The final volume of 20 μl reaction contained 500 ng RNA and real‐time PCR for NF‐160, NGF, and NPY quantification was performed in the CFX96™ Real‐Time System (Bio‐Rad). Data of real‐time PCR were analyzed by the method of 2^−∆∆^ *^C^* ^t^. Results were repeated in two independent experiments, and the assay was run twice for each experiment. The sequences of each primer are listed as follows:

NF160 forward primer: 5′‐GTGGAAGCTCCCAAGCTTAAGGTC‐3′
NF160 reverse primer: 5′‐CTTCTCGGATCCTCCCTCTTCG‐3′
NGF forward primer: 5′‐CCACACTGAGGTGCATAGCGTAAT‐3′
NGF reverse primer: 5′‐CATTGGTAGGATGGGTGGATTCTC‐3′
NPY forward primer: 5′‐CGACAGCATAGTACTTGCCGCC‐3′
NPY reverse primer: 5′‐CATGGGCCTGGAAGTCTAAA‐3′
β‐actin forward primer: 5′‐GCGTGACATTAAGGAGAAGCTGTG‐3′
β‐actin reverse primer: 5′‐TCCACACGGAGTACTTGCGCT‐3′

### 2.6 siRNA transfection

Three groups of SH‐SY5Y cells were transfected with siRNA. First group was transfected with transfection reagent, control siRNA, and DMSO. Second group were transfected with transfection reagent, control siRNA, and CCS‐HE. Third group were transfected with transfection reagent, target NPY siRNA, and CCS‐HE. The transfection procedure was described as follows. Cells were seeded at an initial density of 2 × 10^5^ cells per well in 6‐well plates. After approximately 20 hr, control and target NPY siRNAs as follows (GenScript), and transfection reagent (Invitrogen) were preincubated at room temperature for 15 min in antibiotic and serum‐free media to facilitate formation of lipid‐siRNA complexes. Then, cells were incubated in final transfection mixture containing 0.18% (v/v) Lipofectamine 2000 and 60 nM siRNA in antibiotic and serum‐free medium at 37°C incubator. After 4–5 hr, the medium was replaced with complete medium and gene expression of NPY was assayed after CCS‐HE (0.4 mg/ml) treatment for 24 hr. Transfection conditions were optimized based on maximal gene knockdown as assessed by RT‐PCR.

Control sense siRNA: 5′‐GACACUACAUCAACCUCAUTT‐3′
Control antisense siRNA: 5′‐AUGAGGUUGAUGUAGUGUCTT‐3′
NPY sense siRNA: 5′‐GCCCAUAUUUCAUCGUGUATT‐3′
NPY antisense siRNA: 5′‐UACACGAUGAAAUAUGGGCTT‐3′

### 2.7 Identification of CCS‐HE structures

Twenty microliter of CCS‐HE at 160 mg/ml was chromatographied on an Agilent RRLC system (Agilent Technologies, Inc.) consisting of a ZORBAX SB‐C8 column (5 µm, 4.6 × 250 mm) in a G1316A incubator, a G1312B Dual Unit pump, a G1322A degasser and a G1367D Auto Sample injector with a flow phase consisting of water (95%) and acetonitrile (5%) for 7 min followed by a gradient of acetonitrile (5%–80%) for 5 min at a flow rate of 1 ml/min. Identified single peak signals (Figure 3A) on RRLC chromatography at 210 nm were collected, respectively, according to the retention time, and the collected single fractions were confirmed by chromatography as described above. The confirmed fractions were identified for their masses by Q‐TOF‐MS (LC‐MS) analysis in anion mode. The system consisted of an Agilent 6530 Q‐TOF mass spectrometer (Agilent Technologies Inc.) equipped with an electrospray ionization source (ESI) interface and a MassHunter workstation software version B.02.01 (Agilent Technologies Inc.) containing a compound structure database. The operating parameters were as follows: drying gas (N_2_) flow rate, 10.0 L/min; drying gas temperature, 325°C; sheath gas temperature, 350°C; sheath gas flow rate, 10 L/min; capillary, +3,500 V; fragmentor, 135 V; skimmer, 65 V; collision energy, 20 V; and mass range, 60–400 Da. Standard substances including linolenic acid (LNA), linoleic acid (LA), and oleic acid (OA) (J&K Scientific) were individually chromatographied under same conditions.

### 2.8 Determination of ratio of different UFAs

Standard UFAs including linolenic acid (99.0%), linoleic acid (97.0%), and oleic acid (99.1%) (J&K Scientific) were dissolved in DMSO by ultrasonic to prepare standard samples at various concentrations of 0, 20, 40, 50, 60, 80, and 100 µg/ml for all three standard UFAs. Twenty micro liters of each standard sample was chromatographied on an Agilent RRLC system under same conditions described as above. Signal peak area was linearly regressed with the concentration of standard unsaturated fatty acid to make the standard curve for each unsaturated fatty acid. Same volume (20 µl) of CCS‐HE sample was chromatographied as same as standard UFAs on an Agilent RRLC system, and the area of identical signal peak to each standard unsaturated fatty acid was measured with software on Agilent RRLC system. Individual unsaturated fatty acid content and the ratio in mass of three UFAs were calculated according to the prepared standard curves.

### 2.9 Statistical analysis

Means of measured parameters from each group were analyzed, and standard errors were produced by using statistical software SPSS version 6.1 (StatSoft Inc.) according to manufacturer's instruction. Probability levels of *p* < .05 were considered as statistically significant. Error bars in the graphs indicated standard error (*SE*) of the mean.

## 3 RESULTS

### 3.1 CCS‐HE induced the neurite outgrowth of SH‐SY5Y cells

**FIGURE 1:**
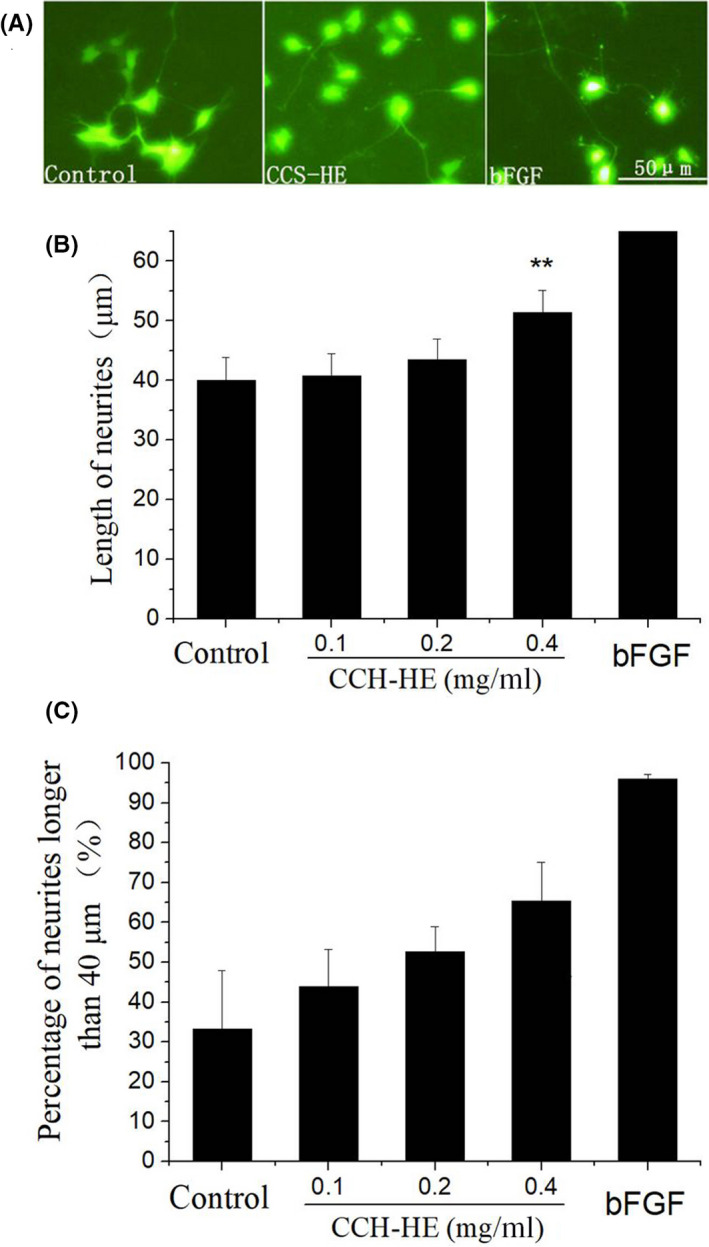
Effects of CCS‐HE on the neurite outgrowth of SH‐SY5Y‐EGFP cells. (A) Neurite outgrowth was induced by CCS‐HE (0.4 mg/ml) and bFGF (20 ng/ml) for 6 days; (B) Longest length of neurite outgrowth from 50 cells was measured in each group. Data represents mean ± *SE* (*n* = 3, **p < .01); (C) Percentage of neurite outgrowth with longer than 40 µm in 50 cells. Data represent mean ± *SE* from three independent experiments (*n* = 3)

To verify the activity of CCS‐HE in neuronal development, CCS‐HE was used to test its effect on the neurite outgrowth of SH‐SY5Y cells. CCS‐HE was added to SH‐SY5Y cell medium for a prolonged 6 days. As shown in Figure 1A, basic fibroblast growth factor (bFGF) or fibroblast growth factor 2 (FGF‐2), a positive control for the induction of neurite outgrowth, induced the neurite outgrowth of SH‐SY5Y‐EGFP cells. As expected, CCS‐HE also induced the neurite outgrowth. Both CCS‐HE and bFGF groups had longer neurite outgrowth than blank group. To quantify the length of neurite outgrowth, six randomly captured images from each group were used to measure the length by the software on the microscope, as described in “Section 2.” As shown in Figure 1B, CCS‐HE at 0.4 mg/ml significantly induced the neurite outgrowth of SH‐SY5Y‐EGFP cells after 6 days while bFGF significantly stimulated the neurite outgrowth. However, low doses (below 0.4 mg/ml) of CCS‐HE did not significantly show the stimulation on the neurite outgrowth. Interestingly, all cells in bFGF group had neurite outgrowth with a neurite length longer than 40 μm and the percentage of neurite outgrowth with longer than 40 μm in all 50 cells measured showed an encouraging response in a dose‐dependent manner (Figure 1C), indicating that CCS‐HE contains bioactive constituents that can stimulate the neurite outgrowth of SH‐SY5Y cells.

### 3.2 CCS‐HE induced NGF, NF160, and NPY expression

**FIGURE 2:**
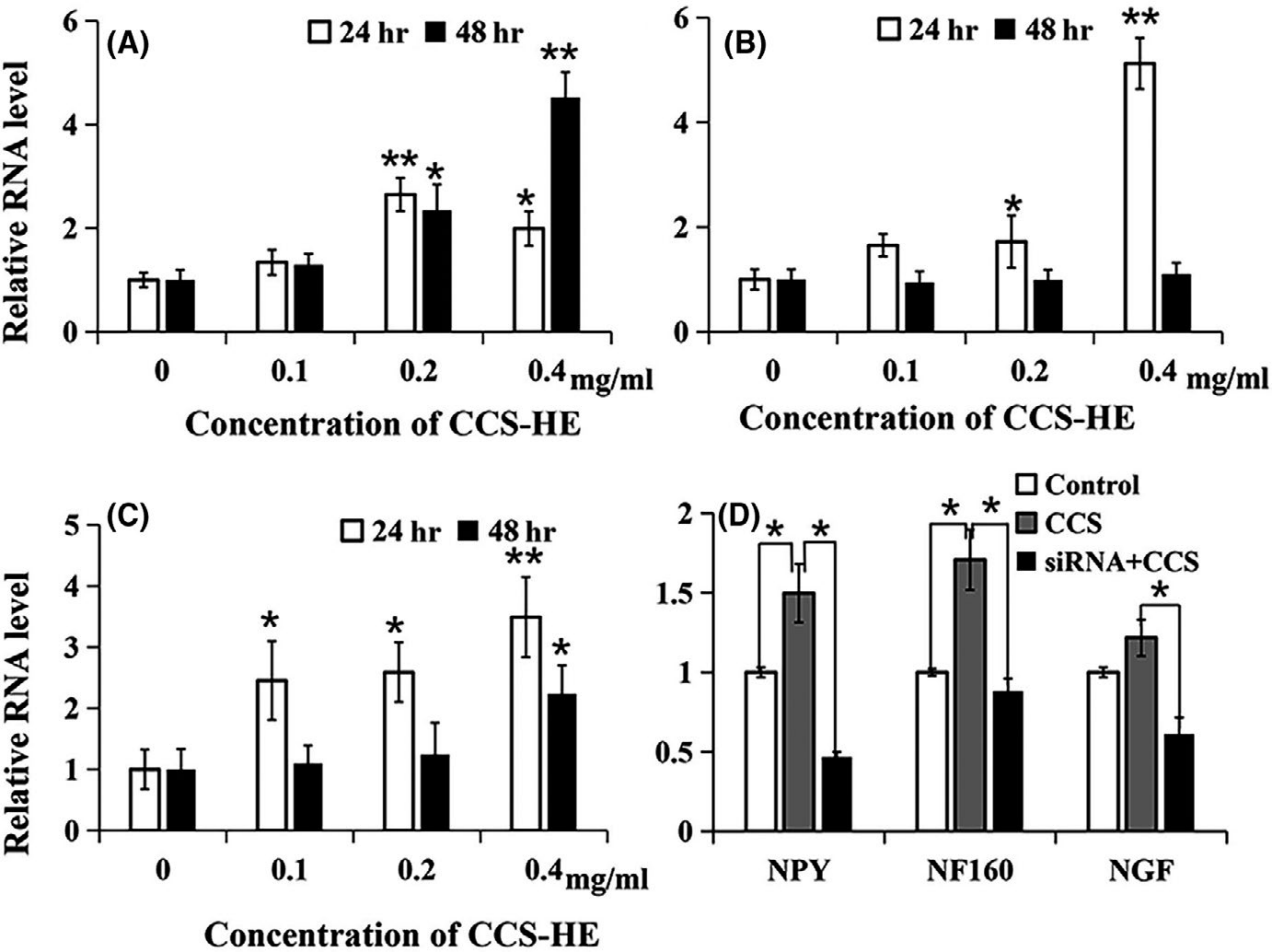
Induction of NF160, NGF, and NPY gene expression by CCS‐HE. (A) NF160 gene expression was induced by CCS‐HE at different doses for 24 and 48 hr; (B) NGF gene expression was induced by CCS‐HE at different doses for 24 and 48 hr; (C) Cells were treated with different doses of CCS‐HE for 24 and 48 hr; (D) Attenuation of CCS‐HE‐induced NGF and NF160 expression by silencing NPY expression. Cells were treated with CCS‐HE at 0.4 mg/ml for 24 hr after NPY siRNA treatment as described in “Section 2.” Data represents mean ± *SE* from three independent experiments (*n* = 3, *p < .05, **p < .01)

In this study, NF160 and NGF were measured as molecular indicators of CCS‐HE effects on neurite outgrowth. As shown in Figure 2A, NF160 expression at mRNA level was significantly upregulated by CCS‐HE in a dose‐dependent manner at 24 and 48 hr. However, NF160 mRNA levels at 48 hr were higher than those at 24 hr, showing an increasing trend in a prolonged treatment. Interestingly, NGF expression was also significantly upregulated by CCS‐HE in a dose‐dependent manner at 24 hr; however, the upregulation was not observed at 48 hr and level of NGF mRNA declined back to control level (Figure 2B), implying a relationship of growth and decline of relative level between NGF and NF160.

To understand whether CCS‐HE regulated gene expression of NGF and NF160 via NPY, a regulator of the upstream gene of the growth factors, regulation of NPY gene expression by CCS‐HE was investigated. As shown in Figure 2C, NPY gene expression was also significantly upregulated by CCS‐HE in a dose‐dependent manner for 24 and 48 hr. However, NPY mRNA levels at 48 hr were lower than those at 24 hr in all three doses of CCS‐HE, showing a decline for a prolonged treatment.

### 3.3 Silencing NPY gene expression attenuated CCS‐HE‐induced NF160 and NGF gene expression

To further confirm whether CCS‐HE increased gene expression of NF160 and NGF via NPY, siRNA was used to reduce NPY gene expression. As shown in Figure 2D, consistent with Figure 2A,B, CCS‐HE significantly upregulated the gene expression of NF160, NGF, and NPY. As expected, NPY siRNA significantly depleted NPY expression induced by CCS‐HE by over 70%. Intriguingly, NPY depletion significantly resulted in attenuation of NGF and NF160 expression induced by CCS‐HE, further supporting an involvement of NPY in the induction of both NGF and NF160 expression by CCS‐HE.

### 3.4 Bioactive constituents were identified as UFAs

**FIGURE 3:**
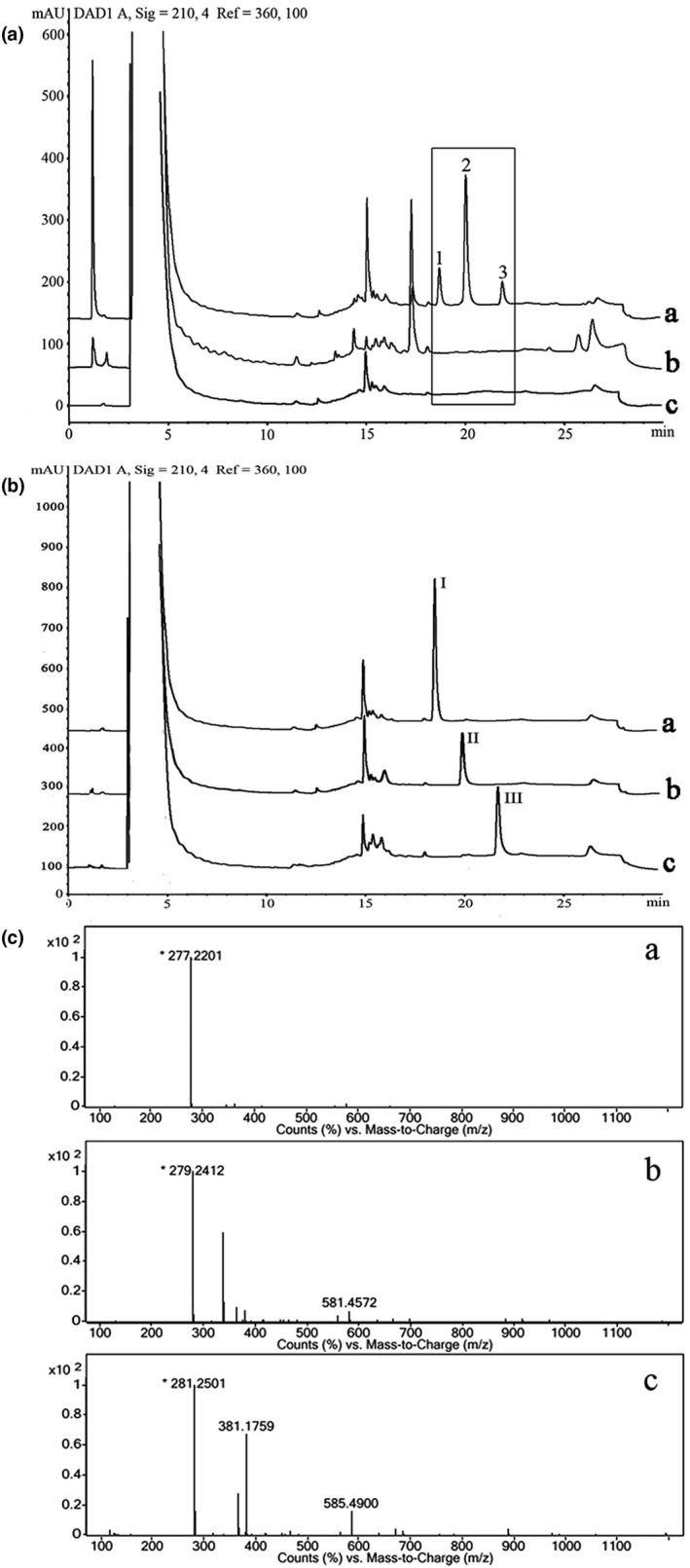
Identification of functional constituents in CCS‐HE. (A) CCS‐HE sample, a, was chromatographied in a flow phase consisting of water and acetonitrile (see “Section 2”). Petroleum ether, b, and DMSO, c, were used as blank controls. Three specific signal peaks at 18.6 min (1), 20.0 min (2) and 21.8 min (3) of retention time were detected at 210 nm. (B) LNA (a), LA (b) and OA (c), were chromatographied by HPLC analysis under same conditions. DMSO (c in FIGURE 3A), was used as a blank control. Three specific signals, peak 1, peak 2, and peak 3, in CCS‐HE were identical to LNA, LA, and OA, respectively. (C) CCS‐HE sample was analyzed by Q‐TOF mass spectrometry, and three masses (277, 281 and 282) were detected in anion mode, which were identical to the masses of LNA (a, 278), LA (b, 280), and OA (c, 282). LA, linoleic acid; LNA, linolenic acid; OA, oleic acid

To identify the constituents of CCS‐HE, which can induce the neurite outgrowth of SH‐SY5Y cells and upregulate gene expression in the process of neurite outgrowth, CCS‐HE was chromatographied by HPLC analysis. As shown in Figure 3A, CCS‐HE contains three featured signal peaks in the box, peak 1 at 18.6 min, peak 2 at 20.0 min, and peak 3 at 21.8 min of retention time (Figure 3A, a), which are absent in petroleum ether used for CCS‐HE extraction (Figure 3A, b) and DMSO used for CCS‐HE dissolvability (Figure 3A, c).

In order to identify the properties of these three signals, next methods were applied for this purpose. First, three signal peaks of CCS‐HE were collected, respectively, and their masses were identified by Q‐TOF mass spectrometry analysis. As shown in Figure 3C, masses of protonized formula of three signals in CCS‐HE are 277 (Figure 3C, a), 279 (Figure 3C, b), and 281 (Figure 3C, c) Daltons in anion mode, respectively. According to database equipped on Q‐TOF mass spectrometry analysis system, top listed structure candidates are identical to masses of LNA (278), LA (280), and OA (282). According to the standard curve of UFA content versus signal peak area, the mass ratio of LNA, LA, and OA was calculated at approximately 1:8:16 and individual contents of three UFAs were 0.096, 0.754, and 1.46 µg/mg, respectively, in CCS‐HE samples. Second, based on Q‐TOF mass spectrometry analysis, the chromatography of these signals was further compared to that of standard substances including LNA, LA, and OA. As shown in Figure 3B, LNA, LA, and OA in DMSO showed a single peak a at 18.6 min (Figure 3B, a), peak b at 20.0 min (Figure 3B, b), and peak c at 21.8 min (Figure 3B, c), respectively, compared to DMSO blank (Figure 3A, c) on HPLC chromatography. Three peaks 1–3 in CCS‐HE (Figure 3A, a) were identical to LNA, LA, and OA, respectively, based on the results of Q‐TOF mass spectrometry and retention time. In the experiments of neurite outgrowth induced by CCS‐HE, individual concentrations of three UFAs were calculated at 0.138, 1.080, and 2.070 µM, respectively, and total concentration of UFAs was 3.288 µM corresponding to 0.4 mg/ml CCS‐HE (Table 1).

**TABLE 1:** Mass ratio and content of UFAs

UFA	Mass Ratio	Content in CCS‐HE (mg/g)	Content in fresh hickory nuts (mg/g)	Concentration in cell cuture (μM)^a^
Linolenic acid	1	0.096	0.048	0.138
Linoleic acid	8	0.754	0.377	1.080
Oleic acid	16	1.460	0.730	2.070
Total	/	2.310	1.155	3.288

^a^ Corresponding to CCS‐HE concentration Hickory Nuts.

### 3.5 Mixture of UFAs induced the neurite outgrowth of SH‐SY5Y cells

**FIGURE 4:**
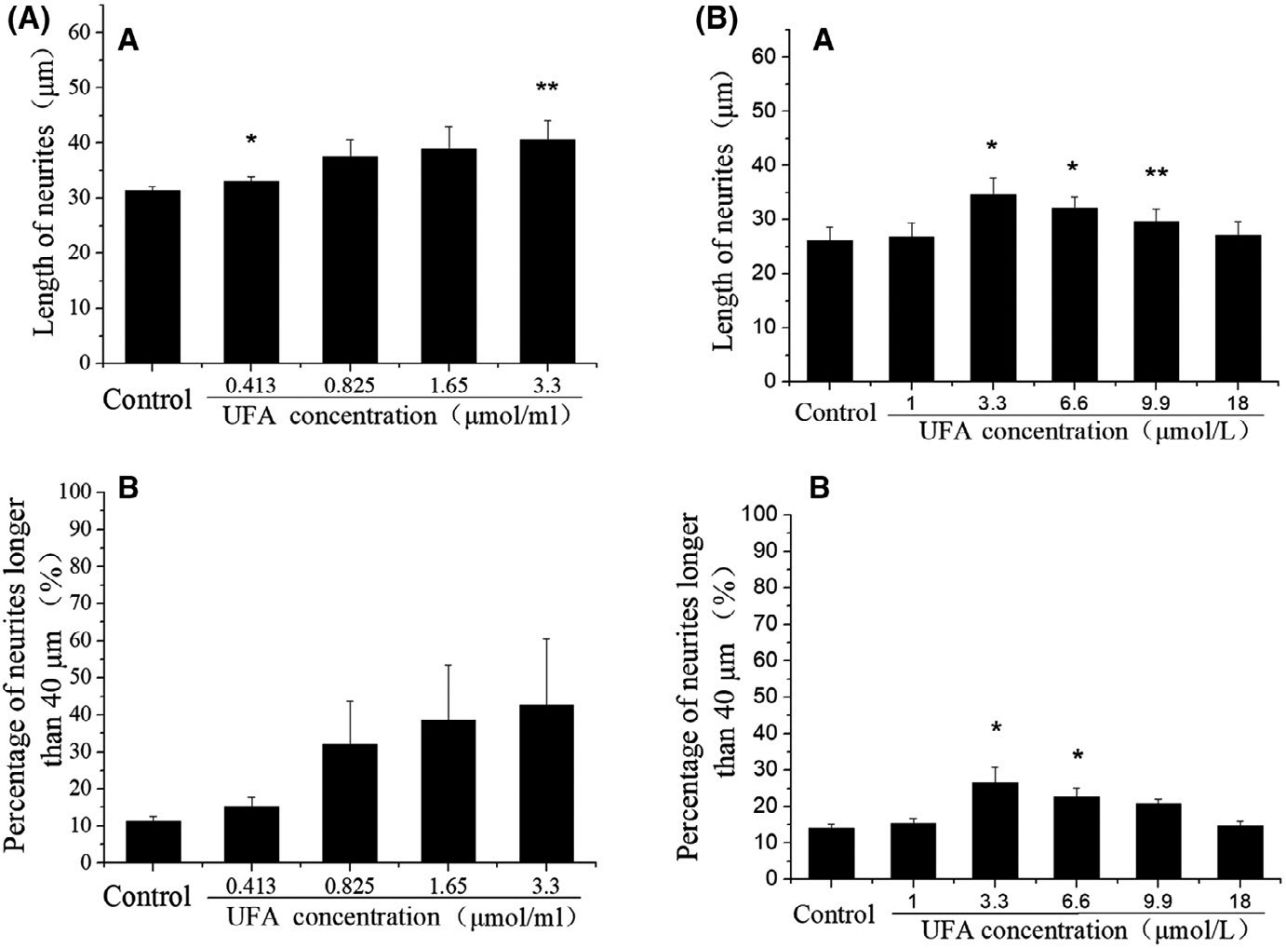
Effects of UFA mixture on the neurite outgrowth of SH‐SY5Y cells. (A) Neurite outgrowth was induced for 6 days by a mixture of LNA, LA, and OA in a mass ratio of 1:8:16 at various concentrations of 0, 0.413, 0.825, 1.65, and 3.30 µM total UFAs corresponding to 0, 0.05, 0.1, 0.2, and 0.4 mg/ml CCS‐HE, respectively. Or (B) higher concentrations of total UFAs in the ratio of 1:8:16. Percentage of neurite outgrowth with longer than 40 µm was counted in 50 cells. Data represent mean ± *SE* from three independent experiments (*n* = 3, **p < .01, *p < .05). LA, linoleic acid; LNA, linolenic acid; OA, oleic acid

**FIGURE 5:**
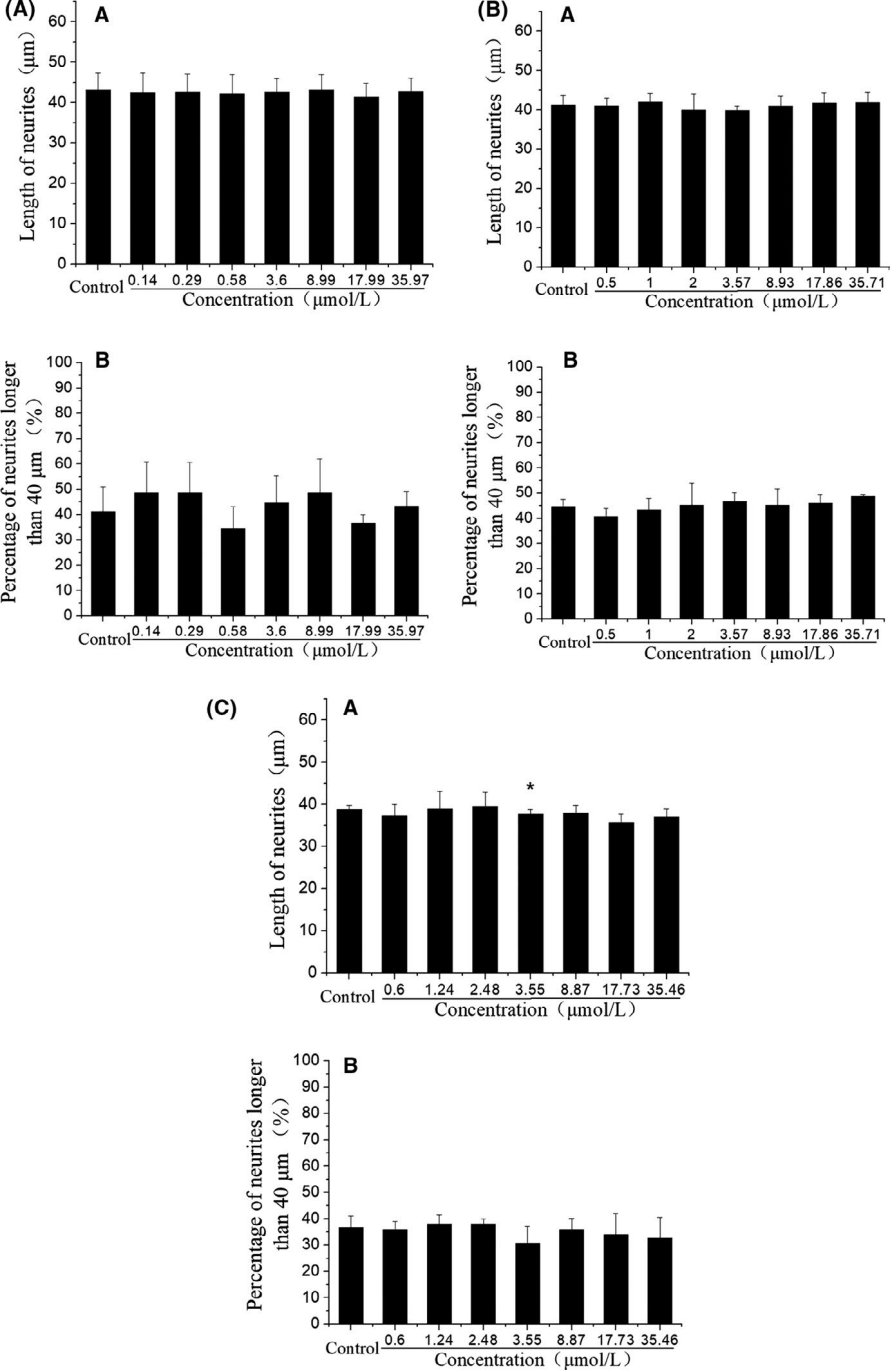
Effects of individual UFA on the neurite outgrowth of SH‐SY5Y cells. Neurite outgrowth was induced for 6 days by LNA (A), LA (B), and OA (C) at various concentrations as indicated. Data represent mean ± *SE* from three independent experiments (*n* = 3, *p < .05). LA, linoleic acid; LNA, linolenic acid; OA, oleic acid

**FIGURE 6:**
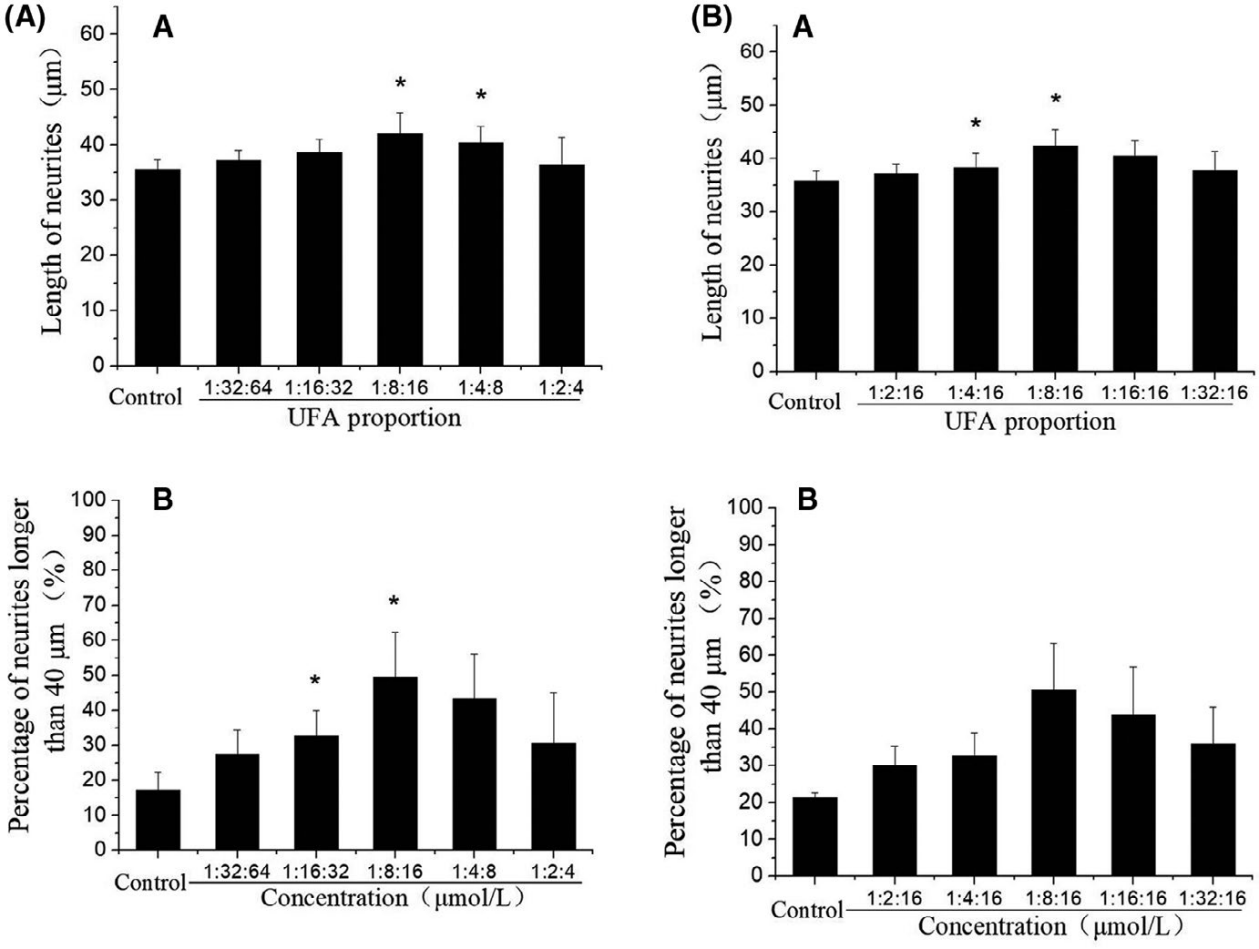
Effects of different ratios of UFAs on the neurite outgrowth of SH‐SY5Y cells. Neurite outgrowth was induced for 6 days by mixtures of linolenic acid, linoleic acid, and oleic acid at a final concentration of 3.30 µM but in various ratios of LNA:LA:OA (A, B) as indicated. Data represent mean ± *SE* from three independent experiments (*n* = 3, *p < .05). LA, linoleic acid; LNA, linolenic acid; OA, oleic acid

Based on the mass ratio of three UFAs in CCS‐HE, a reconstruction of three standard UFAs was prepared to mimic the bioactive constituent of CCS‐HE. As expected, the mixture of UFAs at the mass ratio (1:8:16) induced the neurite outgrowth of SH‐SY5Y cells in a dose‐dependent manner (Figure 4A). Total concentrations of UFAs at 0, 0.413, 0.825, 1.65, and 3.30 µM were corresponded to 0, 0.05, 0.1, 0.2, and 0.4 mg/ml CCS‐HE. The percentage of neurite outgrowth with longer than 40 μm in all 50 cells measured showed a more significant response in a dose‐dependent manner, suggesting that LNA, LA, and OA are bioactive constituents of CCS‐HE. However, further increased concentrations (>3.30 µM) of UFAs showed a decreased tendency of the neurite outgrowth as effective as did 3.30 µM UFAs, showing a best appropriate concentration of total UFAs (Figure 4B). However, we recognized the difference in the percentage of neurite outgrowth with longer than 40 μm in all 50 cells between low and high concentration ranges of UFAs. Also, the individual of each UFA did not show a dose‐dependent manner in the induction of neurite outgrowth and was not effective as observed in the mixture of UFAs even though the concentration of individual UFA was higher than the mixture of UFAs (Figure 5). Further test observed that a natural ratio of LNA:LA:OA, 1:8:16, was a best appropriate ratio to induce the neurite outgrowth (Figure 6).

### 3.6 Mixture of UFAs induced the gene expression of NGF, NF160, and NPY in SH‐SY5Y cells

**FIGURE 7:**
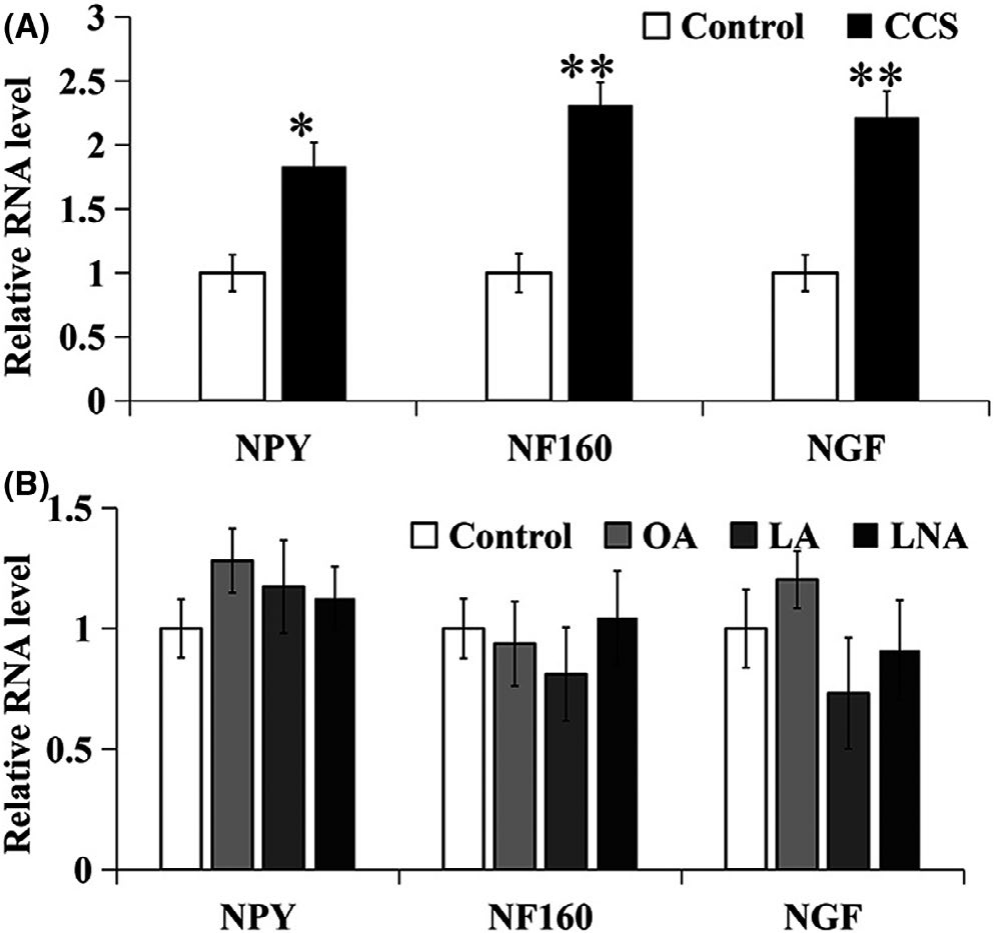
Induction of NF160, NGF, and NPY gene expression by UFAs in SH‐SY5Y cells. (A) mRNA levels of NF160, NGF, and NPY were induced by a mixture of LNA, LA, and OA at a ratio of 1:8:16 and a total concentration 3.30 µM of UFAs, compared to DMSO as a blank control, for 24 hr; (B) mRNA levels of NF160, NGF, and NPY were regulated by the individual of UFAs at 0.138 µM LNA, 1.08 µM LA, and 2.07 µM OA, compared to DMSO as a blank control, for 24 hr. Data represents mean ± *SE* from three independent experiments (*n* = 3, *p < .05, **p < .01). LA, linoleic acid; LNA, linolenic acid; OA, oleic acid

Consistent with that the mixture of UFAs induced the neurite outgrowth (Figure 4), the reconstruction of three UFAs at a final concentration of 3.30 µM significantly upregulated the gene expression of NGF, NF160, and NPY in SH‐SY5Y cells for 24 hr treatment (Figure 7A). However, the individual UFA at 0.138 µM LNA, 1.080 µM LA, and 2.070 µM OA, which consisted of the mixture of three UFAs at 3.288 µM, did not induce the gene expression of NGF, NF160, and NPY (Figure 7B).

## 4 DISCUSSION

Nuts are an important category of food resources for UFA supplies in human nutrition. However, it is rare to investigate that the different ratios of UFAs are relative of their nutritional values in the nervous system. In this study, we demonstrated that CCS‐HE had activity to promote the neurite outgrowth in human SH‐SY5Y cells (Figure 1), supporting CCS as a conventional folk medicine in China for brain health. This activity for the neurite outgrowth of neuronal morphology was supported by which CCS‐HE upregulated the gene expression of NF160 and NGF (Figure 2A,B), one cytoskeleton protein for the neurite outgrowth (Al‐Chalabi & Miller, 2003; Madduri et al., 2009; Reuss, Dono, & Unsicker, 2003) and one neurotrophic factor for stimulating neurite outgrowth (Huang et al., 2010; Schimmelpfeng et al., 2004), respectively. The activity was further supported by which CCS‐HE also upregulated gene expression of NPY (Figure 2C), a modulator of NGF and NF proteins (Angelucci et al., 2014; Croce et al., 2011; White, 1998), and NPY depletion by siRNA attenuated the upregulation of NGF and NF160 expression by CCS‐HE (Figure 2D). The data suggest that CCS‐HE probably exerts a role in neuronal development via turning NPY function on.

In this study, it was observed that CCS‐HE induced the neurite outgrowth in a dose‐dependent manner and further increased dosage tested in the study showed a declined induction to control level (Figure 4). However, sluggish function of CCS‐HE could be amplified via alternative evaluation. The function of CCS‐HE in stimulation of the neurite outgrowth became significant while the neurite outgrowth was evaluated by the percentage of cells with the neurite length longer than a certain length value, for instance, 40 µm in this study, over total cell numbers (Figure 1). Thus, the percentage is a better parameter to evaluate the function of CCS‐HE in stimulation of neuronal development. Both NGF and NF160 expression were upregulated by CCS‐HE; however, they showed different patterns. NGF upregulation by CCS‐HE showed a transient pattern and NF160 did show a prolonged upregulation by CCS‐HE (Figure 2). This is consistent with literature reports that NGF can regulate NF160 expression (Huang et al., 2010; Schimmelpfeng et al., 2004) and NF160 is one of three neurofilaments for the core structures of the neurite outgrowth and axonal elongation (Angelucci et al., 2014; Barnes & Polleux, 2009; Cheng & Poo, 2012). This is also consistent with morphologic change of human SH‐SY5Y cells induced by CCS‐HE even though we did not measure both NGF and NF160 expression at an earlier stage (e.g., 12 hr), suggesting that CCS‐HE stimulated neurite outgrowth by upregulation of NGF expression and NGF further turned on NF160 expression for neurite outgrowth.

Our further study indicated that NPY was also involved in the induction of the neurite outgrowth by CCS‐HE and probably an upstream modulator of NGF and NF160 because CCS‐HE upregulated gene expression of NF160, NGF, and NPY whereas NPY depletion by siRNA attenuated the induced expression of NF160 and NGF by CCS‐HE (Figure 2C,D). Although the upregulated patterns of NPY, NGF, and NF160 expression by CCS‐HE did not show a precise relationship of growth and decline, attenuation of CCS‐HE‐induced NGF and NF160 expression by NPY depletion indicated that CCS‐HE‐induced NPY expression was an upstream event of NGF and NF160 expression in the process of neurite outgrowth by CCS‐HE. These results were consistent with literature reports that NPY is a neurotransmitter and/or a modulator of several neuroendocrine functions to regulate gene expression of NGF and NFs and plays a role in neuroprotection (Colton & Vitek, 2006). More recently, it has been shown that NPY was involved in Alzheimer's disease (AD) and NPY exerted neuroprotective action associated with changes in intracellular production of NGF (Angelucci et al., 2014; Colton & Vitek, 2006; White & Mansfield, 1996). Thus, these data support that CCS consumption is beneficial to brain health, described in ancient Chinese herbal book.

This study further identified that LNA, LA, and OA in an appropriate ratio was a bioactive constituent of CCS‐HE by analysis of HPLC chromatography combined with standard UFAs (Figure 3A,C) and of Q‐TOF mass spectrometry (Figure 3B). The reconstruction of three standard UFAs according to their ratio in CCS‐HE mimicked the constituent of CCS‐HE to induce the neurite outgrowth and gene expression of NGF, NF160, and NPY in SH‐SY5Y cells, and any alternative ratios did not reveal as effective as the natural ratio in the neurite outgrowth and gene expression in human SH‐SY5Y cells (Figures 4, 5, 6, 7), supporting that these three UFAs were ones of bioactive constituents of CCS‐HE in an appropriate ratio. Our studies observed that the individual of UFA at reported dose in literature did not consistently induce the neurite outgrowth (Figure 5). These results are not completely consistent with literature reports that the individual of these UFAs contributed to the neurite outgrowth in neuronal cell line, neuronal cultures, and embryonic chick motoneurons in a range of 10–40 µM (Bento‐Abreu, Tabernero, & Medina, 2007; Darios & Davletov, 2006; Dehaut, Bertrand, Miltaud, Pouplard‐Barthelaix, & Maingault, 1993; Robson, Dyall, Sidloff, & Michael‐Titus, 2010). However, it is consistent with the report that LNA (18:3 *n*‐3) and LA (18:2 *n*‐6) individuals fail to increase mRNA level of growth‐associated protein‐43 (GAP‐43), another marker of axonal growth essentially for the neurite outgrowth, in human SH‐SY5Y cells (Wu et al., 2009).

Our study mainly focused on in vitro cell model and whether CCS‐HE could perform similar effects as that in vivo is unclear. This study provided the information on which human SH‐SY5Y cells could be used as in vitro cell model to reliably evaluate the bioactivity of hickory nut extracts for natural products. It also encourages us to use this in vitro model to continue our work. Future study will further identify the mechanism of UFAs mixture‐induced neurite outgrowth. Furthermore, optimized ratio of different UFAs in in vitro cell model will be verified in model animals for future clinical trial. The long‐term goal for clinical practice of UFAs mixture will be the manipulation of neuronal development and the recovery of neuronal repair for neurodegenerative disorders.

## CONFLICT OF INTEREST

We declare that we have no conflict of interest.

## References

[fsn31623-bib-0001] Al‐Chalabi, A. , & Miller, C. C. (2003). Neurofilaments and neurological disease. BioEssays, 25(4), 346–355. 10.1002/bies.10251 12655642

[fsn31623-bib-0002] Angelucci, F. , Gelfo, F. , Fiore, M. , Croce, N. , Mathe, A. A. , Bernardini, S. , & Caltagirone, C. (2014). The effect of neuropeptide Y on cell survival and neurotrophin expression in in‐vitro models of Alzheimer's disease. Canadian Journal of Physiology and Pharmacology, 92(8), 621–630. 10.1139/cjpp-2014-0099 25026432

[fsn31623-bib-0003] Barnes, A. P. , & Polleux, F. (2009). Establishment of axon‐dendrite polarity in developing neurons. Annual Review of Neuroscience, 32, 347–381. 10.1146/annurev.neuro.31.060407.125536 PMC3170863 19400726

[fsn31623-bib-0004] Bento‐Abreu, A. , Tabernero, A. , & Medina, J. M. (2007). Peroxisome proliferator‐activated receptor‐alpha is required for the neurotrophic effect of oleic acid in neurons. Journal of Neurochemistry, 103(3), 871–881. 10.1111/j.1471-4159.2007.04807.x 17683485

[fsn31623-bib-0005] Boku, S. , Nakagawa, S. , Toda, H. , Kato, A. , Takamura, N. , Omiya, Y. , … Koyama, T. (2013). ROCK2 regulates bFGF‐induced proliferation of SH‐SY5Y cells through GSK‐3beta and beta‐catenin pathway. Brain Research, 1492, 7–17. 23211630 10.1016/j.brainres.2012.11.034

[fsn31623-bib-0006] Cao, X. D. , Ding, Z. S. , Jiang, F. S. , Ding, X. H. , Chen, J. Z. , Chen, S. H. , & Lv, G. Y. (2012). Antitumor constituents from the leaves of *Carya cathayensis* . Natural Product Research, 26(22), 2089–2094. 22007794 10.1080/14786419.2011.628174

[fsn31623-bib-0007] Chen, Z. L. , Yu, W. M. , & Strickland, S. (2007). Peripheral regeneration. Annual Review of Neuroscience, 30, 209–233. 10.1146/annurev.neuro.30.051606.094337 17341159

[fsn31623-bib-0008] Cheng, P. L. , & Poo, M. M. (2012). Early events in axon/dendrite polarization. Annual Review of Neuroscience, 35, 181–201. 10.1146/annurev-neuro-061010-113618 22715881

[fsn31623-bib-0009] Colton, C. A. , & Vitek, M. P. (2006). NPY and chronic neurodegenerative disease. EXS, 95, 223–244. 10.1007/3-7643-7417-9_17 16383010

[fsn31623-bib-0010] Croce, N. , Dinallo, V. , Ricci, V. , Federici, G. , Caltagirone, C. , Bernardini, S. , & Angelucci, F. (2011). Neuroprotective effect of neuropeptide Y against beta‐amyloid 25–35 toxicity in SH‐SY5Y neuroblastoma cells is associated with increased neurotrophin production. Neuro‐Degenerative Diseases, 8(5), 300–309. 10.1159/000323468 21346312

[fsn31623-bib-0011] Darios, F. , & Davletov, B. (2006). Omega‐3 and omega‐6 fatty acids stimulate cell membrane expansion by acting on syntaxin 3. Nature, 440(7085), 813–817. 16598260 10.1038/nature04598

[fsn31623-bib-0012] De Vos, K. J. , Grierson, A. J. , Ackerley, S. , & Miller, C. C. (2008). Role of axonal transport in neurodegenerative diseases. Annual Review of Neuroscience, 31, 151–173. 10.1146/annurev.neuro.31.061307.090711 18558852

[fsn31623-bib-0013] Dehaut, F. , Bertrand, I. , Miltaud, T. , Pouplard‐Barthelaix, A. , & Maingault, M. (1993). n‐6 polyunsaturated fatty acids increase the neurite length of PC12 cells and embryonic chick motoneurons. Neuroscience Letters, 161(2), 133–136. 10.1016/0304-3940(93)90277-R 8272254

[fsn31623-bib-0014] Eipper, B. A. , Stoffers, D. A. , & Mains, R. E. (1992). The biosynthesis of neuropeptides: Peptide alpha‐amidation. Annual Review of Neuroscience, 15, 57–85. 10.1146/annurev.ne.15.030192.000421 1575450

[fsn31623-bib-0015] Freeman, R. S. , Burch, R. L. , Crowder, R. J. , Lomb, D. J. , Schoell, M. C. , Straub, J. A. , & Xie, L. (2004). NGF deprivation‐induced gene expression: After ten years, where do we stand? Progress in Brain Research, 146, 111–126. 14699960 10.1016/S0079-6123(03)46008-1

[fsn31623-bib-0016] Goldberg, J. L. , & Barres, B. A. (2000). The relationship between neuronal survival and regeneration. Annual Review of Neuroscience, 23, 579–612. 10.1146/annurev.neuro.23.1.579 10845076

[fsn31623-bib-0017] Gray, T. S. , & Morley, J. E. (1986). Neuropeptide Y: Anatomical distribution and possible function in mammalian nervous system. Life Sciences, 38(5), 389–401. 10.1016/0024-3205(86)90061-5 3003479

[fsn31623-bib-0018] Huang, E. J. , & Reichardt, L. F. (2001). Neurotrophins: Roles in neuronal development and function. Annual Review of Neuroscience, 24, 677–736. 10.1146/annurev.neuro.24.1.677 PMC2758233 11520916

[fsn31623-bib-0019] Huang, F. , Dong, X. , Zhang, L. , Zhang, X. , Zhao, D. , Bai, X. , & Li, Z. (2010). GM1 and nerve growth factor modulate mitochondrial membrane potential and neurofilament light mRNA expression in cultured dorsal root ganglion and spinal cord neurons during excitotoxic glutamate exposure. Journal of Clinical Neuroscience, 17(4), 495–500. 10.1016/j.jocn.2009.07.112 20171893

[fsn31623-bib-0020] Huang, J. , Zhang, T. , Zhang, Q. , Chen, M. , Wang, Z. , Zheng, B. , … Huang, Y. (2016). The mechanism of high contents of oil and oleic acid revealed by transcriptomic and lipidomic analysis during embryogenesis in *Carya cathayensis* Sarg. BMC Genomics, 17(1), 113 10.1186/s12864-016-2434-7 26878846 PMC4755018

[fsn31623-bib-0021] Loers, G. , & Schachner, M. (2007). Recognition molecules and neural repair. Journal of Neurochemistry, 101(4), 865–882. 10.1111/j.1471-4159.2006.04409.x 17254012

[fsn31623-bib-0022] Madduri, S. , Papaloizos, M. , & Gander, B. (2009). Synergistic effect of GDNF and NGF on axonal branching and elongation in vitro. Neuroscience Research, 65(1), 88–97. 10.1016/j.neures.2009.06.003 19523996

[fsn31623-bib-0023] Martino, M. , Rocchi, G. , Escelsior, A. , Contini, P. , Colicchio, S. , de Berardis, D. , … Fornaro, M. (2013). NGF serum levels variations in major depressed patients receiving duloxetine. Psychoneuroendocrinology, 38(9), 1824–1828. 10.1016/j.psyneuen.2013.02.009 23507186

[fsn31623-bib-0024] Martinotti, G. , Di Iorio, G. , Marini, S. , Ricci, V. , De Berardis, D. , & Di Giannantonio, M. (2012). Nerve growth factor and brain‐derived neurotrophic factor concentrations in schizophrenia: A review. Journal of Biological Regulators and Homeostatic Agents, 26(3), 347–356. 23034254

[fsn31623-bib-0025] Nciri, R. , Bourogaa, E. , Jbahi, S. , Allagui, M. S. , Elfeki, A. , Vincent, C. , & Croute, F. (2014). Chronic neuroprotective effects of low concentration lithium on SH‐SY5Y cells: Possible involvement of stress proteins and gene expression. Neural Regeneration Research, 9(7), 735–740. 10.4103/1673-5374.131578 25206881 PMC4146276

[fsn31623-bib-0026] Qian, Y. , Zheng, Y. , & Tiffany‐Castiglioni, E. (2009). Valproate reversibly reduces neurite outgrowth by human SY5Y neuroblastoma cells. Brain Research, 1302, 21–33. 10.1016/j.brainres.2009.09.051 19766605

[fsn31623-bib-0027] Reuss, B. , Dono, R. , & Unsicker, K. (2003). Functions of fibroblast growth factor (FGF)‐2 and FGF‐5 in astroglial differentiation and blood‐brain barrier permeability: Evidence from mouse mutants. Journal of Neuroscience, 23(16), 6404–6412. 10.1523/JNEUROSCI.23-16-06404.2003 12878680 PMC6740627

[fsn31623-bib-0028] Robson, L. G. , Dyall, S. , Sidloff, D. , & Michael‐Titus, A. T. (2010). Omega‐3 polyunsaturated fatty acids increase the neurite outgrowth of rat sensory neurones throughout development and in aged animals. Neurobiology of Aging, 31(4), 678–687. 10.1016/j.neurobiolaging.2008.05.027 18620782

[fsn31623-bib-0029] Schimmelpfeng, J. , Weibezahn, K. F. , & Dertinger, H. (2004). Quantification of NGF‐dependent neuronal differentiation of PC‐12 cells by means of neurofilament‐L mRNA expression and neuronal outgrowth. Journal of Neuroscience Methods, 139(2), 299–306. 10.1016/j.jneumeth.2004.05.010 15488244

[fsn31623-bib-0030] Sofroniew, M. V. , Howe, C. L. , & Mobley, W. C. (2001). Nerve growth factor signaling, neuroprotection, and neural repair. Annual Review of Neuroscience, 24, 1217–1281. 10.1146/annurev.neuro.24.1.1217 11520933

[fsn31623-bib-0031] Tian, S. S. , Jiang, F. S. , Zhang, K. , Zhu, X. X. , Jin, B. , Lu, J. J. , & Ding, Z. S. (2014). Flavonoids from the leaves of *Carya cathayensis* Sarg. inhibit vascular endothelial growth factor‐induced angiogenesis. Fitoterapia, 92, 34–40. 10.1016/j.fitote.2013.09.016 24096161

[fsn31623-bib-0032] Wen, X. , Xu, S. , Liu, H. , Zhang, Q. , Liang, H. , Yang, C. , & Wang, H. (2013). Neurotoxicity induced by bupivacaine via T‐type calcium channels in SH‐SY5Y cells. PLoS ONE, 8(5), e62942 10.1371/journal.pone.0062942 23658789 PMC3642072

[fsn31623-bib-0033] White, D. M. (1998). Contribution of neurotrophin‐3 to the neuropeptide Y‐induced increase in neurite outgrowth of rat dorsal root ganglion cells. Neuroscience, 86(1), 257–263. 10.1016/S0306-4522(98)00034-7 9692759

[fsn31623-bib-0034] White, D. M. , & Mansfield, K. (1996). Vasoactive intestinal polypeptide and neuropeptide Y act indirectly to increase neurite outgrowth of dissociated dorsal root ganglion cells. Neuroscience, 73(3), 881–887. 10.1016/0306-4522(96)00055-3 8809806

[fsn31623-bib-0035] Wu, H. , Ichikawa, S. , Tani, S. , Zhu, B. , Tada, M. , Shimoishi, Y. , … Nakamura, Y. (2009). Docosahexaenoic acid induces ERK1/2 activation and neuritogenesis via intracellular reactive oxygen species production in human neuroblastoma SH‐SY5Y cells. Biochimica et Biophysica Acta (BBA) ‐ Molecular and Cell Biology of Lipids, 1791(1), 8–16. 18996496 10.1016/j.bbalip.2008.10.004

[fsn31623-bib-0036] Yang, X. , Sheng, W. , Sun, G. Y. , & Lee, J. C. (2011). Effects of fatty acid unsaturation numbers on membrane fluidity and alpha‐secretase‐dependent amyloid precursor protein processing. Neurochemistry International, 58(3), 321–329. 21184792 10.1016/j.neuint.2010.12.004 PMC3040984

